# Vip3C proteins from *Paenibacillus* spp. for controlling lepidopteran crop pests

**DOI:** 10.1128/aem.00253-25

**Published:** 2025-06-20

**Authors:** Todd Ciche, William Moar, Aqeel Ahmad, David Bowen, Catherine Chay, Arlene Howe, Uma Kesanapalli, Jennifer Lutke, Gregory Bean, Jason Milligan, Michael Pleau, Yong Yin, Waseem Akbar, Marty Heppler, Cara Griffith, Kimberly Morrell, Katherine Dunkmann, Heather Anderson, Jeffrey Ahrens, Pacifica Sommers, E. Sethe Burgie, Fred Zinnel, Meiying Zheng, James Fitzpatrick, Michael Rau, Timothy Rydel, Tommi White, David Kerns, James Roberts

**Affiliations:** 1 Bayer Crop Science 733089 , Chesterfield, Missouri, USA; 2 Washington University Center for Cellular Imaging 552102, St. Louis, Missouri, USA; 3 Department of Entomology, Texas A&M University 171804, College Station, Texas, USA; UMR Processus Infectieux en Milieu Insulaire Tropical, Ste. Clotilde, France

**Keywords:** *Paenibacillus*, *Bacillus thuringiensis*, *Ostrinia nubilalis*, transgenic crops, biotechnology, cotton, maize, *Helicoverpa zea*, *Diatraea grandiosella*, *Spodoptera frugiperda*, *Chloridea virescens*, vegetative insecticidal protein, Cryo-EM, protein structure, *Paenibacillus popilliae*

## Abstract

New proteins are needed to control insects not controlled with *Bacillus thuringiensis* (Bt) crops, and those evolving resistance to Bt crops. These proteins are increasingly being reported from non-Bt organisms to control Bt-resistant insects. However, these proteins mostly control the corn rootworm, *Diabrotica* spp. (Coleoptera), whereas most Bt-resistant insects are lepidopteran. We hypothesized that diversifying our search for proteins into non-Bt organisms, such as those related to *Paenibacillus popilliae,* used to control Japanese beetle *Popillia japonica*, could yield proteins with new insecticidal activities against Lepidoptera. Here, we identified Vip3Cb1 and Vip3Cc1 with broad lepidopteran activity, the first Vip3 proteins discovered from strains in the *P. popilliae-*containing clade. Vip3Cb1 protected plants against cotton bollworm, *Helicoverpa zea* and tobacco budworm*, Chloridea virescens* and *H. zea,* fall armyworm, *Spodoptera frugiperda*, and Southwestern corn borer, *Diatraea grandiosella*, in cotton and maize, respectively, like commercial Vip3Aa. Distinct from Vip3Aa, Vip3Cb1 also protected maize against European corn borer, *Ostrinia nubilalis*, the primary maize pest in the United States, with recent reports of resistance to Bt proteins. Consistent with previous reports, insects resistant to Vip3Aa were cross-resistant to Vip3Cb1. Cryo-electron microscopy demonstrated that Vip3Cb1 formed a pore-shaped tetramer upon proteolytic activation, in agreement with the pore-forming mechanism of action of Vip3Aa. Thus, diversifying the search beyond Bt has led to the discovery of the first Vip3 proteins from *Paenibacillus* spp. with different activity spectra from Vip3Aa, providing additional tools to control pests, including those currently resistant to Bt Cry proteins.

**IMPORTANCE:**

New insecticidal proteins are needed for controlling insect pests that can devastate crop yield if left uncontrolled. Diversifying our search for new insecticidal proteins in *Paenibacillus* spp. resulted in the discovery of Vip3Cb1 and Vip3Cc1 insecticidal proteins active against lepidopteran crop pests. Structure and cross-resistance studies indicate overlap in the mechanism of action between Vip3Cb1 and commercial Vip3Aa. However, new activities, such as controlling European corn borer, make these proteins important new tools in the insect control toolbox.

## INTRODUCTION

Crop plants genetically modified to express insecticidal proteins are important for sustainably controlling insect pests (1, 2). *Bacillus thuringiensis* (Bt) insecticidal bacteria have been the predominant source of commercial traits, with diverse proteins controlling Lepidoptera, Coleoptera, and Hemiptera pests (3–7). However, new proteins are needed to control insect pests evolving resistance to commercial Bt crops (8–11) or insect pests not susceptible to the current array of insecticidal proteins in Bt crops. The search for new proteins to control insect pests is increasingly expanding to non-Bt organisms. Most new proteins reported from non-Bt organisms control the corn rootworm, *Diabrotica* spp. (Coleoptera), including *Brevibacillus laterosporus* (12), *Alcaligenes faecalis* (13), *Chromobacterium piscinae* (14), *Pleurotus* spp. (15), *Pseudomonas chloroaphis* (16), and *Ophioglossum pendulum* (17). Although most examples of Bt resistance occur with Lepidoptera (11), the few new proteins reported from non-Bt organisms to control Lepidoptera are primarily from fern species such as *Pteris* (18) and *Adiantum* (19), or *Photorhabdus luminescens* (20, 21). Therefore, the discovery of additional lepidopteran-active proteins from non-Bt sources is critical for effective resistance management and expanded pest range in next-generation insect-protected transgenic crops.

*Paenibacillus popilliae* is the causative agent of milky disease in Japanese beetle, *Popillia japanica* (22), and has been used for biological control since 1939 (23, 24). *P. popillae*, and the closely related *P. lentimorbus*, are primarily known to be pathogenic to beetles (Coleoptera: Scarabeidae) (22). Insect pathogenic *Paenibacillus spp*. are known to contain Cry endotoxins: *P. popilliae* (Cry18) and *P. lentimorbus* (Cry43) (25). We hypothesized that conducting an extensive survey of *Paenibacillus* isolates, some of which are fastidious (22), and screening against various lepidopteran crop pests would result in the discovery of proteins with new lepidopteran activity.

Here we identified Vip3Cb1 and Vip3Cc1, the first Vip3 proteins with broad lepidopteran activity discovered outside of Bt from *Paenibacillus* strains in the *P. popilliae-*containing clade, most closely linked to *P. dendritiformis* (26). Vip3Cb1 and Vip3Cc1 are highly divergent in structural domains known to function in receptor binding and to known insecticidal proteins such as commercial Vip3Aa (27). Vip3Cb1 protected against cotton bollworm, *Helicoverpa zea* and tobacco budworm*, Chloridea virescens*, and *H. zea*, fall armyworm, *Spodoptera frugiperda,* and Southwestern corn borer, *Diatraea grandiosella*, in cotton and corn, respectively, like commercial Vip3Aa. Distinct from Vip3Aa, Vip3Cb1 also protected maize against European corn borer, *Ostrinia nubilalis,* the primary maize pest in the United States, with recent reports of resistance to Bt (28, 29). At least partial cross-resistance was observed when Vip3Aa-resistant fall armyworm, *Spodoptera frugiperda,* and Vip3Aa-resistant *H. zea* were bioassayed using Vip3Cb1, consistent with previous reporting (30). Cryo-electron microscopy (Cryo-EM) demonstrated that Vip3Cb1 formed a pore-shaped tetramer upon proteolytic activation, in agreement with the pore-forming mechanism of action of Vip3Aa. Therefore, the discovery of new Vip3 proteins from *Paenibacillus spp*. has provided important new tools for the control of lepidopteran insect pests.

## RESULTS

### Discovery of *Paenibacillus spp*. isolates

**Fig 1:**
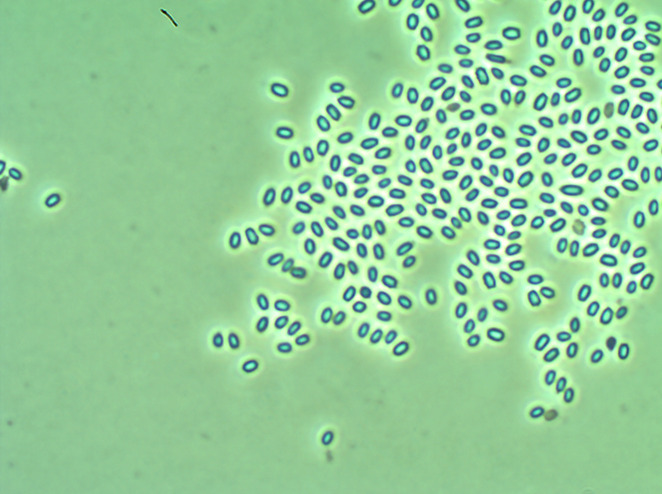
Phase contrast micrograph of *Paenibacillus* strain DSC004343 (Vip3Cb1) endospores.

*Paenibacillus* sp. strain DSC004343 was isolated from an apiary (New Melle, Missouri). Strain DSC020651 was isolated from agricultural soil (Waterman, Illinois). *Paenibacillus* sp. colonies were distinguished from Bt by their slower growth (2–3 d) for colony formation and small colony size relative to Bt. DSC004343 and DSC020651 had cream-colored colonies with smooth margins and produced cells with endospores without parasporal crystal (Cry) proteins visible (Fig. 1).

### DNA sequencing of *Paenibacillus* isolates

**Fig 2:**
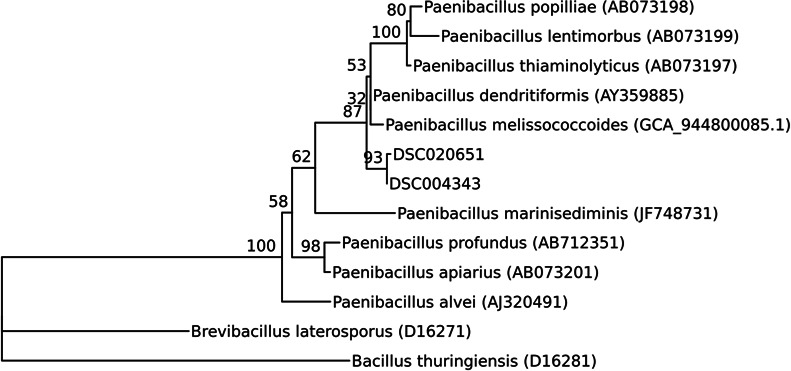
The 16S rRNA genes from *Paenibacillus* strains that produced toxins in a maximum-likelihood phylogeny with similar sequences from the Silva LTP database, *Brevibacillus laterosporus* and *Bacillus thuringiensis* as an outgroup, and the recently published *P. melissococcoides* genome. Values represent bootstrapping support for internal nodes.

DNA sequencing generated 10,529,014 reads for DSC004343 and 10,193,684 reads for DSC020651, resulting in assemblies of 6.60 and 6.45 megabases, respectively. Draft assemblies were >99% complete, with <3% single-copy genes being duplicated, fragmented, or missing (Table S1). 16S rRNA genes identified from assemblies clustered within a well-supported clade with *Paenibacillus popilliae, P. lentimorbus, P. thiaminolyticus, P. dendritiformis,* and *P. melissococcoides* (Fig. 2). DSC004343 and DSC020651 source organisms are more like *P. dendritiformis* (26)*,* than *P. popilliae* (99.2 vs. 98.7% id 16S), or other species within this clade such as *P. melissococcoides* (31) (98.6% similarity). These isolates may be conspecific with *P. dendritiformis*, clustering together 71% of the time (bootstrap), conspecific with distinct species, or constitute novel species. Thus, the taxonomy of these strains is described as *Paenibacillus* spp. in the *P. popilliae-*containing clade.

### Discovery and initial insecticidal activity of Vip3Cb1 and Vip3Cc1 proteins

**Fig 3:**
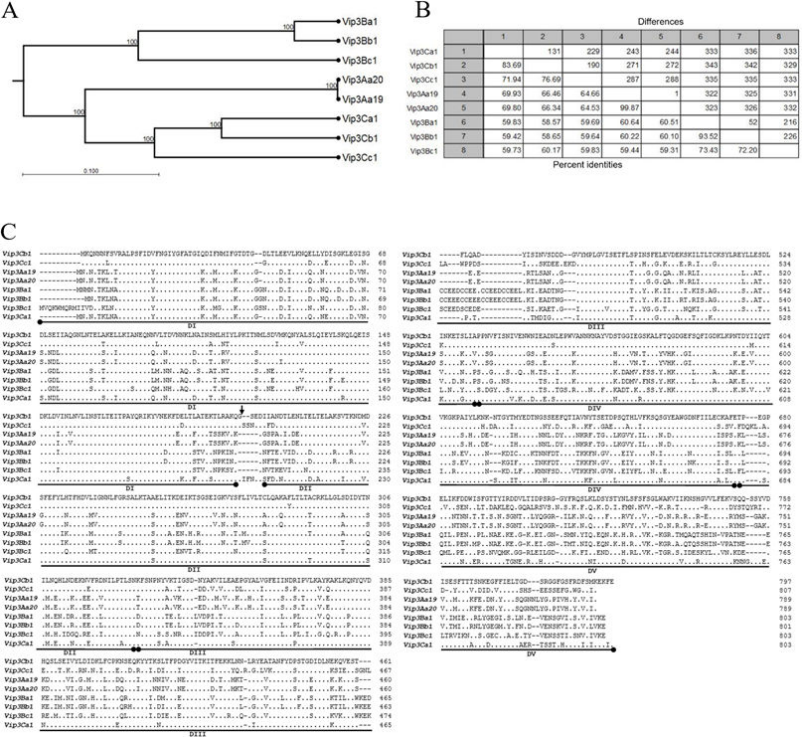
Vip3Cb1 and Vip3Cc1 relatedness to select Vip3 proteins. (**A**) Phylogenetic tree. (**B**) Pairwise comparison with the number of AA differences and percent identities. (**C**) Multiple sequence alignments where domains DI-IV and a predicted proteolytic cleavage site (vertical arrow) are indicated.

Vip3Cb1 and Vip3Cc1 proteins were discovered from whole-genome assemblies of DSC004343 and DSC020651, respectively, based on amino acid homology and conserved protein family domains (32). Amino acid sequences for Vip3Cb1 and Vip3Cc1 were submitted to the Bacterial Pesticidal Protein Resource Center (BPPRC) for classification (32). Sequence alignments demonstrated 83.4% and 76.7% amino acid identity of Vip3Cb1 and Vip3Cc1 to Vip3Ca3, respectively, and 69.8% and 60.0% identity of Vip3Cb1 and Vip3Cc1 to Vip3Aa20, respectively (Fig. 3A and B). Vip3Cb1 and Vip3Cc1 are 76.1% identical to each other. Vip3 proteins, including Vip3Cb1 and Vip3Cc1, are highly conserved in Domains (D) I and II involved in pore formation, with divergence in DIII, IV, and V known to be involved in receptor binding to the gut of the insect host (Fig. 3C). For example, Vip3Cb1 is 79% identical in DI and II and 60% identical in DIII-V to Vip3Aa19 by BLASTP analysis.

Vip3Cb1 and Vip3Cc1 expressed in *E. coli* and Bt demonstrated broad-spectrum activity against numerous lepidopteran crop pests such as *H. zea, O. nubilalis*, *C. includens*, and Southwestern corn borer, *Diatraea grandiosella*, but not against coleopteran insects (Table 1). Vip3Cb1 also displayed insecticidal activity against *C. virescens, S. frugiperda,* and velvetbean caterpillar, *Anticarsia gemmatalis*, activities lacking from Vip3Cc1. The most sensitive species to both proteins tested was *C. includens*. Overall, Vip3Cb1 was more toxic to the species evaluated than Vip3Cc1 and thus, the focus of additional studies reported here.

**TABLE 1:** Stunting and mortality of lepidopteran and coleopteran insects when fed Vip3Cb1 and Vip3Cc1

Organism	VIP3Cb1 Activity score (µg/cm^2^)^*a*^^,^^*b*^	VIP3Cc1 Activity score (µg/cm^2^)^*a*^^,^^*b*^
Lepidoptera		
*Chloridea virescens*	+++ (0.78)	− (39)
*Helicoverpa zea*	+++ (0.39)	+++ (3.9)
*Spodoptera frugiperda*	+++ (0.16)	− (39)
*Chrysodeixis includens*	+++ (0.04)	+++ (0.24)
*Anticarsia gemmatalis*	+++ (0.39)	− (39)
*Spodoptera eridania*	+++ (0.78)	+++ (0.47)
*Ostrinia nubilalis*	+++ (2.0)	+++ (3.9)
*Diatraea grandiosella*	+++ (0.78)	++ (3.9)
*Agrotis ipsilon*	+++ (0.98)	+++ (3.9)
*Striacosta albicosta*	NT	+ (19.6)
Coleoptera		
*Diabrotica virgifera virgifera*	− (39)	− (39)
*Leptinotarsa decemlineata*	− (39)	− (39)
*Diabrotica undecimpunctata howardi*	− (39)	NT

^*a*^ Stunting and mortality when soluble Vip3C proteins are fed to lepidopteran or coleopteran insect larvae. Activity score: −, no observable effect different from the control buffer; +, low stunting where the insect larvae are 50%–90% the size of larvae fed the buffer control; ++, moderate stunting where the larvae are 25%–50% the size of larvae fed the buffer control and/or <50% mortality; +++, strong stunting where the larvae are <25% the size of larvae fed the buffer control and/or >50% mortality; NT, not tested.

^*b*^ Minimum concentration (µg/cm^2^), where this activity score was observed.

### Vip3Cb1 structure

**Fig 4:**
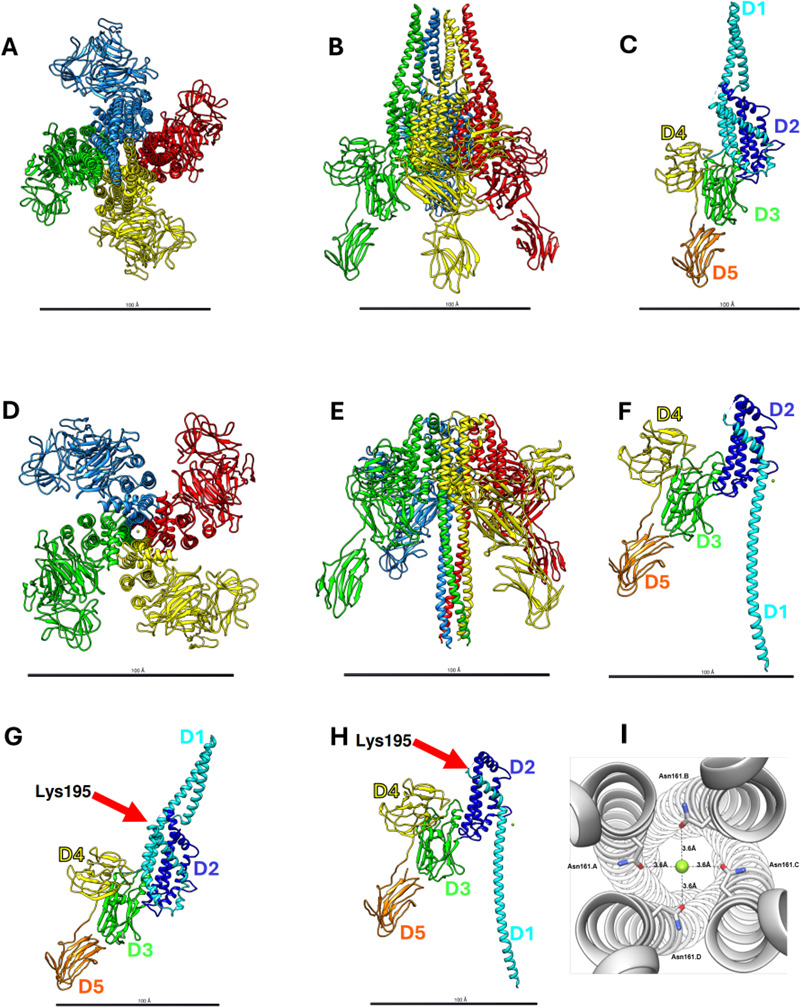
Structures of Vip3Cb1. (**A**) Cryo-EM structures of Vip3Cb1 protoxin (**A through C**) and activated toxin (**D through F**) tetramer top (**A, D**) and side views (**B, E**) and monomer side view showing domain architecture (**C, F**) with D1 in cyan, D2 in blue, D3 in green, D4 in yellow, and D5 in orange. Red arrows indicate where the trypsin activation occurs and subsequent conformational changes in DI-DII, trypsin activation site (Lys195) in the protoxin (**G**), and cleaved after trypsinization (**H**). (**I**) Mg^2+^ (lime green sphere) binding by Asn161 side chain carbonyl (stick, 3.6 Å) in Vip3Cb1 activated toxin viewed from top (scale: 100 Angstroms).

Vip3Cb1 protoxin and trypsin-processed Vip3Cb1 toxin structures were determined using Cryo-EM, resulting in density maps with an estimated resolution of 2.6 Å and 3.0 Å, respectively (Table S2). The protoxin quaternary structure reveals a twofold symmetrical tetramer, composed of a dimer (consisting of monomers A/B or C/D) of dimers (Fig. 4A through C; Fig. S2 and S3, note: RMSD values in S2). Each dimer pair (monomers A/B, or monomers C/D) is conformationally distinct, with equivalency between monomers A and C and monomers B and D. Domains I (DI) (residues 1–192) and II (residues 202–329) are α-helical, whereas DIII (residues 330–533), DIV (residues 534–675), and DV (residues 676–797) are composed of β-sheets, with homology to carbohydrate binding motifs connected by extended loops (Fig. 4G). DI of each monomer is composed of two twisted α-helices (α2 and N-terminal α3) connected by a five amino acid loop, allowing α2 to fold back to interact with α3. In the tetramer, DI folds into what could resemble the nose of a spaceship, with the monomers arranged in an A-B-D-C configuration. DII forms the spaceship main body (α1, C-terminal α3, α4-α9), containing a central helix (α8) surrounded by other helices, preceded by the tryptic site (Lysine195) for protoxin activation (Fig. S1); however, this site had poor density for modeling (residues 195–202). DIII (β1-β14) forms the spaceship base, ending with a helix (α11) that interacts with DII. From DIII, a linker extends, interacting with N-terminal residues, before starting the extended fins of the spaceship, DIV (β15-β24) and DV (β15-β34).

Activation of the Bt Vip3A protoxin occurs by cleavage between domains DI and DII (33, 34). The Vip3Cb1 N-terminus has the signature Vip3A signal peptide “ALPSF” motif (34, 35). Upon activation with trypsin, cleavage occurs preferentially at Lysine 195 (Fig. S1); yet, the tetramer stays intact and undergoes large conformational changes, revealing a pore structure as determined by Cryo-EM (Fig. 4D through F; Fig. S2 and S4). Although the extreme N-terminus density is lacking in the Cryo-EM density maps, modeling from Leu98 in DI is possible. The protoxin’s DI and N-terminal DII unfurl and rearrange into an extended 4 α-helical channel (α1_act_), while domains DIII-DV remain unchanged (Fig. 4H). The former protoxin α10 helix h2 leading into α5 (residues E205–V220) shifts down, becoming a more ordered α3_act_ upon activation. In addition, protoxin α4 (residues 165–192) also moves down, becoming part of the newly formed channel α1_act_. At Pro168, there is a 63^o^ bend in α1_act_ which leads into the long-extended 4α-helical channel. Inside the channel, there was a magnesium ion coordinated to the Asn161 side-chain carbonyl (Fig. 4I), with coordination lengths (3.6 Å consistent with a magnesium hexahydrate ion (36), which was the only divalent ion present in the sample buffer. Structural studies of Vip3Cc1 using both X-ray protein crystallography and Cryo-EM were unsuccessful.

### Transgenic plant efficacy and cross-resistance

**Fig 5:**
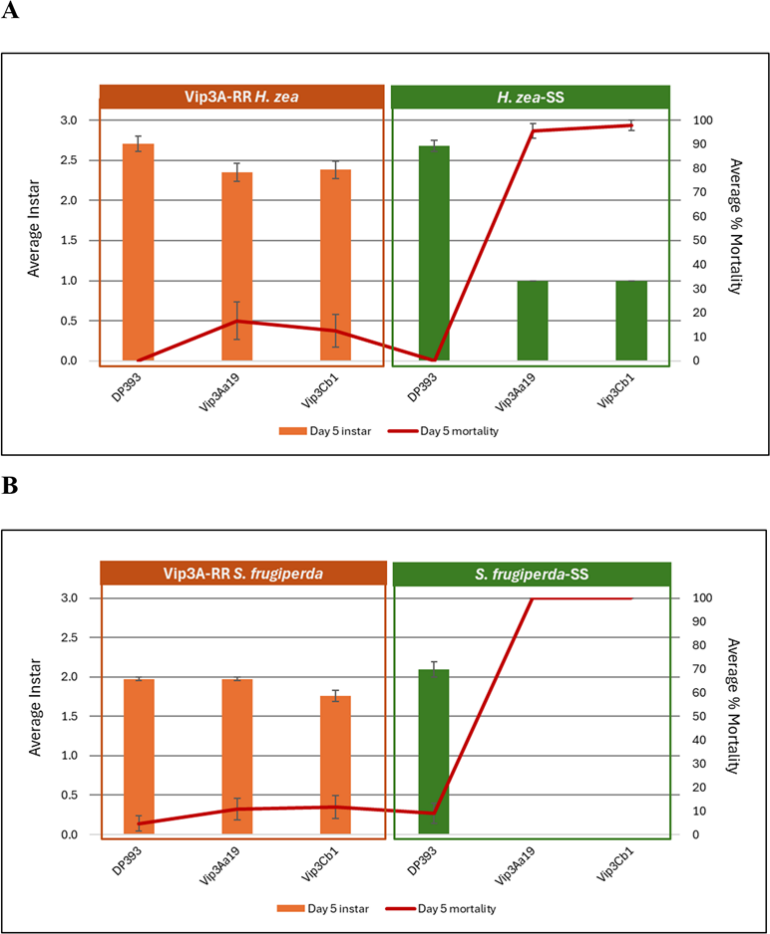
Cross-resistance of Vip3Cb1 to (**A**) Vip3Aa-resistant *Helicoverpa zea* (RR) and (**B**) *Spodoptera frugiperda* (RR) based on average instar and mortality on cotton. DP393 was used as the non-traited control. Susceptible insects for both species were listed as SS. Day 5 instar (bars, left Y-axis) and Day 5 mortality (lines, right Y-axis) are indicated.

Bioassay results in cotton for *H. zea* (Fig. 5A) and *S. frugiperda* (Fig. 5B) demonstrated considerable efficacy of Vip3Aa19 and Vip3Cb1 against susceptible insects, but little activity when these same proteins were tested against Vip3Aa-resistant *H. zea* and *S. frugiperda*. These results confirmed the presence of Vip3Aa resistance alleles in these colonies and at least partial cross-resistance between Vip3Aa and Vip3Cb1.

**Fig 6:**
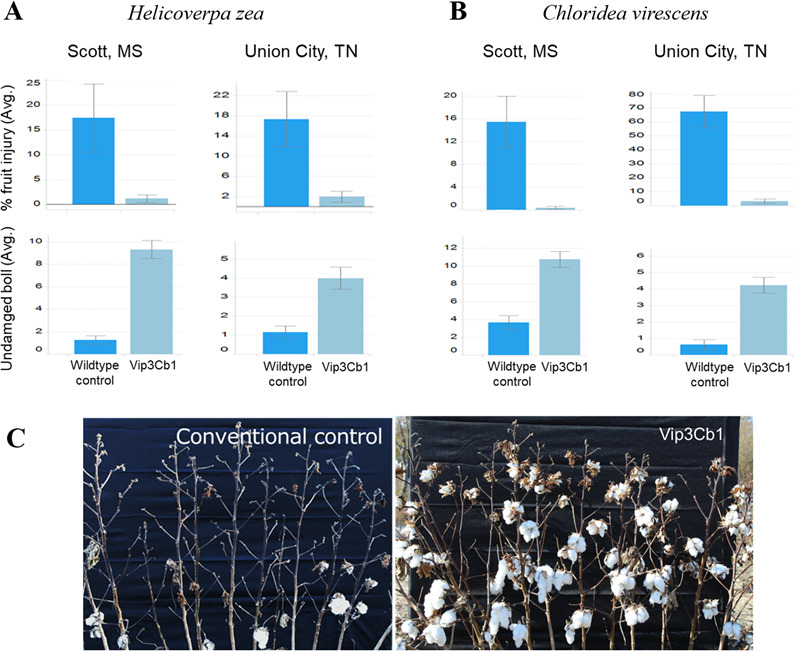
Fruit injury and boll retention of transgenic cotton expressing Vip3Cb1 in screenhouse trials at two locations (**A**) *Helicoverpa. zea* and (**B**) *Chloridea virescens*. (**C**) Lint production in cotton expressing Vip3Cb1 under artificial *H. zea* infestations. DP393 was used as the wild type and the conventional control.

A transgenic cotton event expressing Vip3Cb1 provided fruit protection against *H. zea* and *C. virescens* in screenhouse studies at two locations. Fruit injury levels varied from 15% to nearly 70% in non-transgenic plots, whereas they remained below 5% in Vip3Cb1 plots (Fig. 6A and B). There was a >3-fold increase in undamaged bolls and considerable lint production in Vip3Cb1 plots compared to non-transgenic plots (Fig. 6A through C).

**Fig 7:**
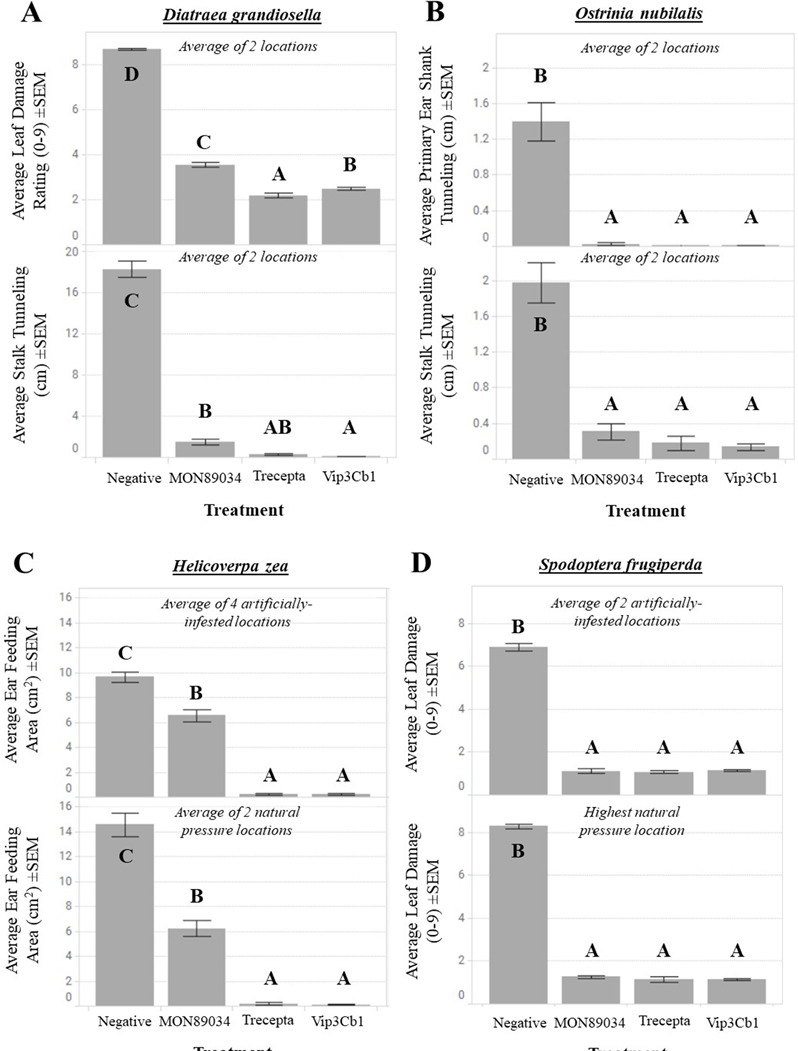
Field efficacy of Vip3Cb1 against key pests: *Diatraea grandiosella* (**A**), *Ostrinia nubilalis* (**B**), *Helicoverpa zea* (**C**), and *Spodoptera frugiperda* (**D**) in artificially infested and natural pressure maize trials. All Vip3Cb1 treatments were significantly (*P* < 0.05) different from the negative control. MON89034 and Trecepta were evaluated as commercial controls.

Field trials were conducted to evaluate efficacy against *H. zea*, *O. nubilalis*, *D. grandiosella*, and *S. frugiperda* in transgenic maize lines expressing Vip3Cb1 fused with a chloroplast-targeting peptide (CTP) in its N-terminus (Fig. 7). Maize-expressing Vip3Cb1 had significantly less ear feeding from *H. zea* than wild type (natural pressure: F_3,144_ = 454.76, *P* < 0.00001, artificial infest: F_3,203_ = 690.19, *P* < 0.00001), less stalk and shank tunneling from *O. nubilalis* than wild type (Stalk: F_4,107_ = 52.09, *P* < 0.00001; Shank: F_4,107_ = 43.26, *P* < 0.00001), less leaf and stalk tunneling damage from *D. grandiosella* than wild type (Leaf: F_4,171_ = 1138.54, *P* < 0.00001; Stalk: F_4,171_ = 681.59, *P* < 0.00001), and less leaf damage from *S. frugiperda* than wild type in both natural pressure (F_3,48_ = 1780.21, *P* < 0.00001) and artificially infested (F_4,123_ = 1393.56, *P* < 0.00001) field trials.

## DISCUSSION

### Discovery

New proteins are needed to control additional insect crop pests not currently controlled with Bt crops, or to control insect crop pests with evolving resistance to Bt crops. We hypothesized that diversifying our search for insecticidal proteins to non-Bt, bacteria such as those closely related to *P. popilliae,* used as a biopesticide for the control of Japanese beetle *P. japonica*, could yield proteins with new lepidopteran activities. Using whole-genome sequencing, we discovered Vip3Cb1 and Vip3Cc1; the first Vip3 lepidopteran-active proteins outside of Bt from *Paenibacillus* strains in the *P. popilliae-*containing clade. The *P. popilliae*-containing clade has the potential to deliver even more new insecticidal proteins in the future because more novel strains like DSC004343 and DSC020651 exist in soil.

### Insecticidal activity of Vip3Cb1 and Vip3Cc1 proteins

Vip3 (Vip3A) proteins were first reported in 1996 (4) and expressed in commercialized transgenic crops in the United States for controlling insect pests of cotton and maize since 2009 (27). There is considerable interest in using Vip3 proteins to control lepidopteran pests, as insects resistant to the traditional Bt Cry proteins are still susceptible to Vip3 proteins (27). Numerous Vip3 proteins have been reported, representing Vip3A, Vip3B, and Vip3C subfamilies (27, 35, 37, 38). Vip3Aa proteins have a broad spectrum of lepidopteran activity, providing commercial-level activity in transgenic crops against many major insect pests such as *H. zea*, *H. armigera*, and *S. frugiperda,* but not *O. nubilalis*. To date, only two Vip3 (Vip3Aa) proteins have been commercialized in insect-protection crops. Several reasons for this include the following: (i) Cross-resistance between Vip3A proteins and between Vip3A and Vip3C proteins, possibly due to shared binding sites (27, 30, 38, 39), although there has only been one case of Vip3A resistance in which resistance was due to an alteration in binding (27), and (ii) Vip3 proteins reporting toxicity against *O. nubilalis* have reduced toxicity against other target insect pests (35, 37). In this study, we have demonstrated Vip3Cb1 as the first Vip3C protein protecting against main Lepidoptera pests, including not only *H. zea*, *S. frugiperda,* and *D. grandiosella* but also *O. nubilalis,* when expressed in transgenic crops (Fig. 5 to 7). In addition, plant efficacy was also evident when Vip3Cb1 was fused with a chloroplast-targeting peptide (CTP) at its N-terminus (Fig. 7), which was designed to target Vip3Cb1 to the chloroplast for possible increased accumulation in plant cells and reduction of potential off-types (40, 41). These data suggest multiple options for optimal plant expression in the development of future Vip3Cb1-containing commercial traits.

There was cross-resistance between Vip3Aa and Vip3C, including Vip3Cb1, as reported here and elsewhere (30). As Vip3Cb1 only shares 69.8% overall homology to Vip3Ab1, and Vip3Ca2 only shares partial receptor binding with Vip3Aa16 (39), cross-resistance would not necessarily be expected based on alteration of binding alone, as typically is the case for Bt proteins such as Cry1A and Cry1C (27). However, the overall mode of action of Vip3 proteins requires multiple steps, many of which, if altered, could confer reduced susceptibility or resistance (27).

As Vip3Aa has been widely used against major resistance risk pests such as *S. frugiperda,* there is concern about Vip3A resistance developing in these pests; therefore, the use of Vip3Cb1 against these same populations where Vip3A has been used extensively should be evaluated with caution. However, with insects in which Vip3Aa is not active commercially (e.g., *O. nubilalis*), Vip3Cb1 would have considerable value, especially in the case of O. *nubilalis,* where this pest recently has been reported to have field-evolved and practical resistance to Bt Cry proteins (28, 29).

Like Cry proteins within the same group, such as Cry1 proteins with lepidopteran activity, Vip3 proteins exhibit considerable differences in their host spectrum. As previously stated, Vip3Aa proteins have wide commercial-level lepidopteran pest activity, with at least the exception of *O. nubilalis*. Vip3Bc1 has *O. nubilalis* activity but does not have *H. zea* or *S. frugiperda* activity (35). Vip3Ca3 is active against many lepidopteran pests but has little to no *S. frugiperda* or *O. nubilalis* activity (42). Vip3Cb1 has activity against *H. zea*, *S. frugiperda*, and *O. nubilalis,* and Vip3Cc1 is active against many lepidopteran pests but has no *S. frugiperda* activity (this study). Future studies should include investigations into the sequences or domains responsible for these differences in activity.

### Structure

Cryo-EM structural data revealed high structural homology between Vip3Cb1-derived *Paenibacillus* spp. and *B. thuringiensis*-derived Vip3Aa, Vip3Bc1, and Vip3B2160 protoxin and Vip3Aa and Vip3Bc1 trypsin-activated toxin forms (Fig. 4; Table S2) (43–45). Comparisons of available Vip3 structures, determined by either X-ray protein crystallography, Vip3B2160 (45) or Cryo-EM Vip3Aa16 (43) and Vip3Bc1 (44), revealed that the Vip3Cb1 protoxin has an overall similar quaternary “spaceship” structure (44) with α-helical DI-DIII forming the body and β-sheet DIV-V forming the fins (Fig. 4; Fig. S2; Table S2). Artificial trypsin digestion mimics Vip3 activation by serine proteases abundant in lepidopteran midgut (34, 46, 47). The activated Vip3Cb1 quaternary structure is also like activated structures determined for Vip3Aa16 and Vip3Bc1 (Fig. S2), with large conformational changes in DI-II forming a long, extended α-helical channel. Vip3Cb1_act_ tryptic site boundary modeling revealed density for the N-terminal 20 kDa fragment up to Asp185, and density resumed for the C-terminal 65 kDa fragment at Asn209, containing DII-V. Detection of a magnesium ion in the Vip3Cb1 channel is evidence that Vip3Cb1 can function as a pore for ions, causing osmotic imbalance and insect cell death (28). This magnesium ion was also observed in the Vip3Aa16_act_ structure (43), similarly coordinated by the Asn (N163) side chain with a similar bond length (3.6 Å), and this Asn residue is highly conserved in Vip3 sequences (44). Therefore, structural comparisons of Vip3Cb1 to other Vip3 proteins, including commercial Vip3Aa, are consistent with other results, such as amino acid homology, that all Vip3 proteins share the same mechanism of action, even though these originated from a *Paenibacillus* strain and not from Bt.

In summary, we discovered the first Vip3 proteins from *Paenibacillus* spp. strains in the *P. popilliae-*containing clade. Vip3Cb1 and Vip3Cc1 displayed new insecticidal spectra to major crop pests. Resistant insect and structural studies reveal overlapping mechanisms of action of Vip3Cb1 to commercial Vip3Aa, limiting the utility of Vip3Cb1 against Vip3A-resistant insects. However, the new insecticidal spectra of Vip3Cb1 and Vip3Cc1 could enable the development of next-generation transgenic crops for protection against several crop pests for which new tools are hard to come by. Therefore, these studies provide further evidence for the strategy of diversifying the search for insecticidal proteins beyond Bt. Diversifying efforts like these and others are likely to continue to be important for the discovery of new insecticidal proteins, which can sustainably protect crops from numerous pests continually evolving resistance to commercial traits.

## MATERIALS AND METHODS

### Isolation and growth of *Paenibacillus* strains

An amount of 0.5–2 g soil from an apiary (New Melle, Missouri) or an agricultural field (Waterman, Illinois) was suspended in 10 mL of 0.85% NaCl for 1 hour at 300 rpm in a sterile flask, then heated at 80°C for 20 min and plated on NYSM (48), supplemented with thiamine (0.02 g), biotin (2.0 µg), and 0.1% sodium pyruvate to enhance growth of *Paenibacillus* (22). Isolates were picked and grown in Terrific Broth (Invitrogen, Waltham, MA) supplemented with thiamine, biotin, and sodium pyruvate.

### Phylogeny construction by 16S rRNA analysis

Genomic DNA was purified as described previously (12) and was sequenced as 2 × 200 base-pair reads on an Illumina HiSeq at >150× coverage based on estimated 6.5 Mb genomes (49, 50). Reads were assembled using SPAdes v1 (51), and the quality of each genome was assessed using BUSCO v 5.50 with default settings and automatic lineage detection for prokaryotes (52).

A phylogenetic tree was constructed based on the 16S hypervariable region of the small subunit ribosomal RNA gene, which was extracted from each assembled genome using barrnap v.0.9 (53). 16S rRNA genes were aligned against the Silva NR99 LTP 12 2021 database using Nucleotide BLAST 2.6.0+ and all matches more than 95.5% similar over at least 70% of the sequence selected, including *B. thuringiensis* and *B. laterosporus* as outgroups. Finally, we downloaded the recently published genomes of *P. melissococcoides* from NCBI Genbank (accessions GCA_944800085.1, GCA_945318265.1, and GCA_945318285.1) and extracted the 16S rRNA genes using barrnap. We then aligned these sequences using cmalign from INFERNAL v.1.1.4 (54), trimmed the ends based on manual inspection to a sequence length of 1,493 nt, and built a maximum likelihood tree using IQTree v1.6.1 with 1,000 iterations of bootstrapping support for internal nodes and *B. thuringiensis* designated as the outgroup (55). These commands were run in R v.4.0.3 (56) and the plot visualized with package ggtree v.3.5.0.900 (57).

### Identification

Full-length genes encoding Vip3Cb1 and Vip3Cc1 were putatively identified in draft genome assemblies based on pfam analysis and blast hits to known insecticidal proteins. PCR primers were designed based on the first 30 nucleotides of the 5′ end and the last 30 nucleotides of the 3′ ends of the coding region to generate PCR products. An additional 16 nucleotides were added to both 5′ and 3′ ends to enable hot fusion cloning (41). PCR products were generated using standard PCR conditions with KOD Hot Start DNA polymerase (Novagen, San Diego, CA). The same DNAs used for whole-genome sequencing were used to generate PCR products for hot fusion. PCR products were cloned into a *Bt* expression/*E. coli* shuttle vector (designed and created at Bayer Crop Science). Both genes were subcloned into *E. coli* strain DH10B. Plasmid DNA from *E. coli* clones containing Vip3Cb1 and Vip3Cc1 was isolated and sequence-confirmed by Sanger sequencing (ABI 3700). The two confirmed protein sequences were submitted to the BPPRC and assigned the Vip3Cb1 and Vip3Cc1 designations, respectively (33).

### Expression for insect diet bioassay testing

Bt expression plasmids for Vip3Cb1 and Vip3Cc1, bulked up in *E. coli,* were transformed by electroporation into an atoxigenic *Bt* expression strain for protein production (12, 45). The Bt expression strain may not be optimal for Vip3 secretion, and significant Vip3 protein remained in the cell-associated fraction. Bt expression strains were selected on LB plates with 5 µg/mL chloramphenicol, and colonies were picked for protein expression in C2 liquid culture medium with 5 µg/mL chloramphenicol. Cultures were incubated at 28°C for 3–4 days in 1× C2 medium (1 L: 3.11 g KH_2_PO_4_, 4.66 g K_2_HPO_4_, 2.0 g peptone, 2.0 g yeast extract, 2.0 g (NH_4_)_2_SO_4_, 5.0 g casamino acid, 10.0 g glucose dissolved in 900 mL ddH_2_0, and 100 mL 10× C2 salts composed of 3.0 g MgSO_4_.7H_2_O, 1.0 g CaCl_2_.2H_2_O, 0.58 g MnCl_2_.4H_2_O, 0.050 g ZnCl_2_.7H_2_O, 0.050 g CuSO_4_.5H_2_O, 0.005 g, and FeSO_4_.7H_2_O) and assessed for sporulation. Insoluble cellular material and spores were harvested by centrifugation at 8,000 × *g*. The supernatant was discarded and washed in 10 mM Tris-HCl, pH 8; 0.005% Triton X-100 and 0.1 mM EDTA (pH 8) (TX), and the cell pellet was resuspended in TX at 1/10 the volume of the original culture and stored at 4°C. The insoluble protein in the washed crystal spore preparation was solubilized by resuspending the washed cell pellet in 25 mM carbonate buffer (pH 10) and 5 mM dithiothreitol (DTT) in 1/10 the original volume, followed by centrifugation at 8,000 × *g* to pellet any remaining insoluble debris. The supernatant was collected and stored at 4°C. The protein quantity was determined by spot densitometry analysis of SDS-PAGE on a Bio-Rad Gel Doc EZ system.

Genes encoding Vip3Cb1 and Vip3Cc1 were cloned into a pET vector containing an in-frame N-terminal his-tag and transformed into *E. coli* Rosetta DE3 cells for protein production. Cells were grown in auto-induction media at 37°C, and the N-terminal his-tag protein was purified using batch nickel chromatography and buffer-exchanged into 25 mM carbonate buffer (pH 10) and 5 mM DTT. Proteins estimated to be greater than 90% pure (by SDS-PAGE) were quantified by Bradford assay and stored at −80°C.

### Insect diet bioassays

All insects were evaluated at one to multiple concentrations as high as 39 µg/cm^2^ Vip3Cb1 and Vip3Cc1 in a diet overlay. Diet bioassays were completed in triplicate.

### Lepidopteran diet overlay bioassays

#### Artificial diet production

All lepidopteran assays were conducted in 96-well plates filled with 200 µL of artificial diet per well. Diet was produced in 2.3 L batches by the addition of 33.3 g Serva agar and 379.5 g of Southland multiple species diet with mold inhibitor to 2.1 L of autoclaved distilled/deionized water. The artificial diet was stored at 4°C.

### Manual infest

Soybean looper, *Chrysodeixis includens* (Bayer Crop Science), black cutworm, *Agrotis ipsilon* (Bayer Crop Science), and tobacco budworm, *C. virescens* (Benzon Research, Carlisle, PA), eggs were received the week of use and held at 15°C. Eggs were incubated at 27°C, 60%–70% of humidity for ~2 days until egg hatch. Insects were transferred to a diet containing 96-well plates previously overlaid with protein test samples. Plates were sealed with pre-perforated heat seals, incubated at 27°C and 60%–70% of humidity for 4–5 days, and scored for stunting and mortality as described below.

### Automated infest

For *H. zea* (Benzon Research, Carlisle, PA), *S. frugiperda* (Benzon Research, Carlisle, PA), *O. nubilalis* (Bayer Crop Science), and D. *grandiosella* (Bayer Crop Science), eggs were received the week of use and held at 15°C until used. Egg sheets were incubated at 27°C with 60%–70% humidity until hatch. Larvae were dispensed into 96-well diet-containing plates previously overlaid with protein by automated liquid transfer (58). Plates were then sealed with pre-perforated heat seals, incubated at 27°C and 60%–70% of humidity for 4–5 days, and scored for stunting and mortality as described below.

### Diet bioassay scoring

Scoring of all diet assays was completed in a column-wise manner. Sample columns were visually compared to the control H_2_O column individually. Activity scores were determined at all concentrations tested as the visual difference in size between the sample column and the H_2_O column. Activity scores were rated on a (−), (+), (++), or (+++) scale. Activity scores of (−), (+), (++), and (+++) were defined as no observable difference in size between the sample column and the H_2_O column, a 25%–50% difference in size, a 50%–75% difference in size and/or <50% mortality, and a >75% difference in size and/or >50% mortality, respectively. The lowest concentration at which this activity score was observed is provided in Table 1. There were 24 wells for each sample. The assay was only valid if it met quality control measures.

### Coleoptera diet overlay bioassays

Western corn rootworm, *D. virgifera virgifera*, Colorado potato beetle, *Leptinotarsa decemlineata*, and Southern corn rootworm, *Diabrotica undecimpunctata howardi*, were tested in diet overlay bioassays with Vip3Cb1 and Vip3Cc1 as previously described (12).

### Vip3Cb1 cloning, expression, and purification for structure determination

Vip3Cb1 was expressed in BL21-codonPlus (DE3)-RIPL *E. coli* strain (Stratagene) using an auto-induction media supplemented with 100 µg mL^−1^ kanamycin and 34 µg mL^−1^ chloramphenicol as described by Studier (59). For the protoxin structure, two cysteine mutations (Cys162 and Cys164) were engineered to disable the toxin from transitioning into the activated form. Proteins were expressed and purified using nickel affinity chromatography followed by size exclusion chromatography. All purification steps were carried out at 4°C. Sub-cultures were grown at 37°C for 3 h, and then grown at 20°C overnight. Cells were harvested by centrifugation at 10,000 × *g* for 10  min and frozen at −80°C. Cell pellets were lysed for 30 min at 4°C with a 3:1 (vol/vol) mixture of B-PER (Bacterial protein extraction reagent, ThermoFisher Scientific) and Y-PER (Yeast protein extraction reagent, ThermoFisher Scientific) supplemented with 25 mM Tris-HCl, 300 mM NaCl, 10 mM imidazole and adjusted to pH 8. The suspension was allowed to proceed for 20 min on ice. Lysate was clarified by centrifugation at 15,000 × *g* for 20  min. Cleared supernatant was loaded onto a 5 mL Ni-NTA column connected to AKTA Pure (Cytiva). The column was washed with 25 mM Tris-HCl, 300 mM NaCl, 30 mM imidazole, and pH 8. Protein was eluted with 25 mM Tris-HCl, 300 mM NaCl, 150 mM imidazole, and pH 8. Relevant protein fractions were pooled, concentrated, and subjected to size-exclusion chromatography using a Superdex 200 10/30G column (Cytiva) equilibrated with 50 mM Tris-HCl pH 8, 500 mM NaCl, 5 mM MgCl2, and 2 mM DTT. Fractions containing pure Vip3Cb1 were pooled and concentrated to 3 mg/mL for negative staining and data collection. Protein concentrations were determined spectrophotometrically based on calculated molar absorption coefficients at 280 nm.

To prepare the trypsin-treated Vip3Cb1 toxin sample, 500 µL Vip3Cb1 (at 2  mg ml^−1^) was incubated with 0.01 mg of L-1-tosylamido-2-phenylethyl chloromethyl ketone (TPCK)-treated trypsin (ThermoFisher Scientific), for 2 hours at room temperature and then subjected to size-exclusion chromatography using a Superdex 200 10/30G column equilibrated with 50 mM Tris-HCl pH8, 500 mM NaCl, 5 mM MgCl2, and 2 mM DTT. Fractions containing pure trypsin-processed Vip3Cb1 were pooled and concentrated to 4.5 mg/mL for data collection.

### Vip3Cb1 limited trypsin digests and mass spectrometry

To identify potential trypsin cleavage sites required for activation of Vip3Cb1, 0.5 mg·ml^−1^ of Vip3Cb1 was incubated with 0.01 mg·ml^−1^ trypsin in 100 mM NaCl and 20 mM EPPS, and pH 8.5. The reaction was conducted at 37°C, quenched at the indicated times by adding 15 µL of the reaction solution to 15 µL of 2X Laemmli sample buffer (Bio-Rad), and boiling for 1 minute. Samples were separated by SDS-PAGE and visualized with Imperial protein stain (ThermoFisher). Gel bands were manually excised, and established sample processing procedures were followed to conduct mass spectrometry. Chymotryptic (Promega Mass spec-grade) digests (10 µL) were injected for nano LC-MS, and data were analyzed using PEAKS and Proteome Discoverer 2.5, disabling enzyme specificity rules and allowing software to match all peptides present.

### Vip3Cb1 Cryo-EM sample preparation and data acquisition

Vip3Cb1 protoxin and trypsin-processed Vip3Cb1 toxin samples were vitrified on Quantifoil Cu 2/2 300-mesh grids, which were freshly plasma-cleaned for 1 min using a Gatan Solarus 950 (Gatan, Pleasanton, CA). An amount of 3 µL of 2 mg/mL sample was deposited on grids and vitrified in liquid ethane by plunge freezing using a Vitrobot Mark IV (ThermoFisher) CryoEM. Vitrobot settings were as follows: chamber at 4°C, 100% humidity with a blot force of −2, blot time of 2 seconds, and a wait time of 20 seconds. Vitrified grids were then imaged on a Cs-corrected ThermoFisher Titan Krios G3 cryo-electron microscope (ThermoFisher Scientific, Eindhoven, NL) operating at 300 kV. Automated data collection was performed using EPU (ThermoFisher), and images were acquired with a Falcon4 direct electron detector (ThermoFisher) operating in counted mode. Each data set was acquired with a nominal magnification of 59 Kx with a resulting pixel size of 1.081 Å, defocus range of −1 to −2.4 µm. For Vip3Cb1 protoxin, 4,547 micrographs were acquired with a total dose of 57.6 e/Å2 with a total exposure time of 13.29 seconds, fractionated over 50 frames. For Vip3Cb1 toxin, 5,644 micrographs were acquired with a total dose of 53.3 e/Å2 with a total exposure time of 13.37 seconds, fractionated over 46 frames.

### Image processing

Both data sets were processed using cryoSPARC v4.3.1 (Punjani, A. & Fleet). Preprocessing was conducted with patch motion correction and patch CTF estimation. Templates were made for Vip3Cb1 protoxin using 1,000 randomly selected micrographs and generated from an ab-initio density, which was used to template pick the entire data set using cryoSPARC’s template picker. Cryo-EM workflows are presented in Fig. S3 and S4. This resulted in 2,763,759 particles extracted with a 320-pixel box. These particles were split into three subsets for 2D classification, and the best classes were combined (1,852,879 particles) into a heterogeneous job using the three ab-initio densities as inputs. One class (1,557,854 particles) aligned to high resolution and was moved to homogeneous refinement applying C2 symmetry, resulting in GSFSC resolution of 2.61 Å. Domains IV and V in this density were not well aligned. To perform local refinement, a mask was created around DIII-DV. These masks were made using UCSF Chimera (v. 1.17.1) (60) by aligning the Vip3Aa model 6TFJ to the density and utilizing the volume zone to isolate the density of DIII-DV (residues 326–789) for both monomers of dimer A/B. These densities were then converted into masks using cryoSPARC volume tools. Local refinement of domains III-V for both chain A and chain B resulted in a resolution of 2.85 Å and 2.82 Å, respectively.

For Vip3Cb1 toxin, templates for template picking were generated from ab initio density that represented Vip3Cb1 toxin. This resulted in 2,758,038 particles being extracted with a box of 450 pixels downsampled to a 200-pixel box. These particles were split into three subsets for 2D classification, and the best classes were combined (407,762 particles) into a heterogeneous job using ab-initio density as inputs. A mixture of Vip3Cb1 protoxin and Vip3Cb1 toxin was separated. Vip3Cb1 toxin class (219,620 particles) was reextracted to a 450-pixel box and used in a homogeneous refinement applying C4 symmetry and subsequent non-uniform refinement, resulting in a resolution of 3.04 Å. DIV and DV in this density were not well aligned like Vip3Cb1 protoxin. Symmetry expansion using C4 symmetry was performed prior to local refinement. To achieve better alignment during local refinement of particles, subtraction of domains DIII-DV of one protomer was performed on the symmetry-expanded data set. The masks for domains DIII-DV and particle subtraction were generated by the same process described above. Subtracted particles were then used for local refinement of domains DIII-V, resulting in a resolution of 3.47 Å.

### Model building/refinement

Initial models for both Vip3Cb1 protoxin and Vip3Cb1 toxin were generated using the Chainsaw program (CCP4) (61) from PDB coordinates of Vip3Aa protoxin (PDB: 6TFJ) and Vip3Aa toxin (PDB: 6TFK) and ClustalW format sequence alignments. These models were aligned to their respective maps using UCSF Chimera (60). For both Vip3Cb1 protoxin and Vip3Cb1 toxin, the main map was used to generate the complete structure of domains I-III, which was subjected to multiple rounds of manual refinement using COOT (v. 0.9.8.5 EL) (61) and real space refinement in Phenix (v. 1.20.1) (62). For the refinement of DIV and DV of chain A/B of Vip3Cb1 protoxin, a composite map was made using Chimera. DIV and V for chain A and B were then subjected to multiple rounds of manual refinement in COOT and real space refinement in Phenix. The refined DIV and DV were then appended to the refined DI-III for chain A/B. NCS symmetry operators were used to generate the full-length tetramer. A single round of ADP refinement was done to the complete tetramer using the main map. The Vip3Cb1 toxin was refined in a similar fashion without the need for a composite map. DIV and DV were refined using the local refinement map and were then appended and refined as described for Vip3Cb1 protoxin. All rendering and structure analysis were performed using UCSF Chimera (v.1.16) (60).

### Generation of transgenic plants expressing Vip3Cb1 in cotton and maize

The Vip3Cb1 coding sequence was redesigned for optimal expression in plants with a targeted GC content of 57% (GenBank accession number PP436397). Each binary vector containing the DNA cassette to drive the expression of Vip3Cb1 was cloned with an additional CP4-EPSPS selection cassette providing resistance to glyphosate for maize, and the SPC/STR cassette to confer spectinomycin and streptomycin resistance in cotton.

For the expression of Vip3Cb1 in cotton, the construct includes a promoter, 5′ leader, and intron of an Actin 2 gene from *Arabidopsis thaliana* and a 3′ UTR of an untranslated region of a seed maturation protein, MP21, from *Medicago truncatula*. The construct was transformed into the cotton line DP393-0053 using *Agrobacterium*-mediated transformation. Single-copy events were selected based on molecular assays and self-pollinated through two generations to produce the R2 progeny used for further studies. Upon visual inspection, no abnormal phenotypes were observed in any transgenic cotton plants expressing Vip3Cb1.

For the expression of Vip3Cb1 in maize, a nucleotide sequence encoding a chloroplast-targeting peptide (SETit.MDH) from *Seteria italica* was fused to the N-terminal region of the redesigned Vip3Cb1 coding sequence. The corn expression construct includes the promoter, 5′ leader, and intron (P-Sb.Ubq7, L-Sb.Ubq7, I-Sb.Ubq7; 2,003 bp upstream of the transcription start site) of a polyubiquitin protein from *Sorghum bicolor* and the 3′ UTR (T-Sb.Nltp4) from a non-specific lipid-transfer protein from *Sorghum bicolor*. The construct was transformed into the maize inbred line LH244 using *Agrobacterium*-mediated transformation. Single-copy events were selected based on molecular assays and grown to produce hybrid F1 seeds by transferring pollen from transgenic plants to donor ears in the 93IDI3 inbred line for further studies. Upon visual inspection, no abnormal phenotypes were observed in any transgenic maize plants expressing Vip3Cb1.

### Evaluation of cross-resistance

Cotton leaf disk bioassays were conducted to evaluate the level of cross-resistance between Vip3Aa and Vip3Cb1 by comparing responses of both Vip3A-resistant and susceptible *H. zea* and *S. frugiperda* colonies (Texas A&M University) to Vip3Aa, Vip3Cb1, or a wild-type control (DP393; Deltapine). Leaf disks were cut from young cotton leaves and placed singly in bioassay tray wells containing 500 µL of 2% agar. Each leaf disk was then infested with one neonate larva from one of the following four insect colonies: Vip3Aa-resistant *H. zea* (Vip3Aa-RR CBW), susceptible *H. zea* (CBW-SS), Vip3Aa-resistant *S. frugiperda* (Vip3Aa-RR FAW), or susceptible *S. frugiperda* (FAW-SS). This process was repeated until sample sizes of 24 for Vip3Aa-RR CBW and 48 for CBW-SS, Vip3Aa-RR FAW, and FAW-SS were achieved. Bioassay trays were then sealed and incubated at 27°C (60% RH and 12:12 photoperiod) for 5 days. Larvae were evaluated for mortality and development (instar) after the experiment. 

### Cotton screenhouse trials

All trials were conducted at Union City, TN, and Scott, MS, locations during the summer, 2019. Treatments consisted of DP393 (Deltapine) as wild-type or conventional control, and Vip3Cb1 expressing cotton. At each location, test blocks were established for infestation by selected target lepidopteran pests. Test plots were planted inside large screenhouses (SH) (30 × 90 = 2,700 square feet) that were artificially infested with moths from laboratory colonies of *H. zea* (CW1802) and *C. virescens* from Bayer Crop Science (Union City, TN). Three thousand moths per species were released in each screenhouse at the early bloom stage (~50–55 days after planting) when there were several matchheads to pre-candle squares and few flowers. Success of the first infestation was assessed after 7–10 days by checking for eggs and larvae in the non-transgenic plots. If needed, a second infestation was conducted with a similar number of moths as the first infestation.

Evaluations were performed mid- to late bloom (~3 weeks after the adult infestation), targeting five plants in each plot. Plants were thoroughly inspected, and the numbers of injured and uninjured squares and bolls were recorded separately for each plant. Injury is defined as partial or complete penetrative damage in the fruiting structures, and not just etching. The percent fruiting injury was calculated based on these numbers.  Plots were not harvested for yield; however, defoliant was applied, and end-of-season pictures were taken to assess potential yield differences.

### Maize field trials

Field trials evaluated the efficacy of Vip3Cb1 events against *H. zea*, *O. nubilalis*, *D. grandiosella*, and *S. frugiperda* that received artificial infestations of insects at six trial locations (two sites in Union City, TN; Paragould, AR; Jonesboro, AR; Thomasboro, IL; and Jerseyville, IL). All insects used in these trials came from Bayer Crop Science. In addition, events were evaluated under natural pressure from wild *H. zea* and *S. frugiperda* populations at three trial locations (Fairhope, AL; Belvidere, NC; Portland, TX).

The two AR locations were irrigated using a drip irrigation system, while the other trial locations were rainfed. Trials were planted between May 11 and June 1, 2017, at optimal windows with weather and in accordance with standard geographical recommendations for these areas. Seed was treated with Acceleron as a fungicide; weed management was entirely by hand. At each location, Vip3Cb1 efficacy was compared with a conventional corn entry as a negative control and positive controls (MON89034, Trecepta, SmartStax). Entries were mapped in the field in a randomized complete block design, with three replicate plots per entry (10′ per row plus 2.5′ alley, 25 seeds/plot, 6″ seed spacing, 30″ row spacing). One-row plots were planted for artificially infested trials, and 4-row plots for natural pressure trials. For the artificial infestations and evaluations, 10 plants per plot were selected for evaluation. Due to low pressure, the Paragould, AR location was not scored for ECB damage, and the Belvidere, NC location was not scored for FAW damage. Plants were artificially infested at appropriate growth stages and evaluated using methodology appropriate to each insect species as described below:

For *D. grandiosella*, freshly hatched (<16 h old) neonates were suspended in corn cob grit, and 30–35 insects were infested into the whorl of each maize plant at V7-V8 and the leaf axil of the primary ear at R2. Fourteen days after the V7-V8 infestation, damage was scored on infested plants using the 0–9 Guthrie Leaf Damage Rating Scale. 27–30 days after R2 infestations, tunneling length (centimeters) in the stalk (from one node above the primary ear down to the crown), as well as the number of tunnels per plant, were evaluated. A trial was considered successful if at least a leaf damage rating of 6 and at least 10 cm of stalk tunneling on negative controls was achieved.

For *O. nubilalis*, freshly hatched (<16 h old) neonates were suspended in corn cob grit, and 70–100 insects were infested into the whorl of each maize plant at V9-V10 and the leaf axil of the primary ear at R2. 27–30 days after R2 infestations, we evaluated tunneling length (centimeters) in the ear shank of the primary ear, tunneling length (centimeters) in the stalk (entire length of the plant from tassel to base), and number of tunnels per plant. A trial was considered successful if an average of at least 5 cm of stalk tunneling on negative controls was achieved.

For *H. zea*, shoot bags with both ends open were placed over the developing primary ear, and plants were infested with fabric squares containing 50–60 blackhead eggs per plant at R1. Approximately 21 days after infestation, the shoot bag was removed, and the feeding area was measured (cm^2^). A trial was considered successful if at least 6 cm^2^ of ear feeding area on negative controls was achieved.

For *S. frugiperda*, freshly hatched (<16 hours old) neonates were suspended in corn cob grit, and 40 insects per plant were infested into the whorl at V6 (63). 14 days after infestation, leaf damage was evaluated using the 0–9 Davis Leaf Damage Rating Scale. A trial was considered successful if a leaf damage rating of at least six on negative controls was achieved.

For natural pressure trials, 0–9 Davis Leaf Damage Ratings were taken at V4, V6, V8, and VT to evaluate *S. frugiperda* efficacy, but the maximum rating was used in the analysis. In addition, at R4, the ear feeding area (cm^2^) was evaluated for *H. zea*.

For all field trials, ANOVAs with treatments nested inside location and experimental replicate were used, followed by a two-sided test of equivalence with *P* < 0.05 (R; the R Project for Statistical Computing). In equivalence tests, treatments with the same letter have differences < 0.05. *P* values were adjusted with a Sidak adjustment to account for one-sided inferences.

## Data Availability

Gene sequences are available in GenBank under the accession numbers LQ835767 for Vip3Cb1 and PP942172 for Vip3Cc1. The protein sequences have been submitted to BPPRC (32). The 16S rRNA genes for the two strains are available in GenBank under accession numbers PQ614276 for strain DSC004343 and PQ614277 for DSC020651. The protein structure Cryo-EM maps are deposited in the electron microscopy databank, and protein structure models are uploaded to the Protein Data Bank (VIP3Cb1 Protoxin: EMDB EMD-47974 and PDB 9EFI, VIP3Cb1 Toxin: EMDB EMD-47972 and PDB 9EFG) (64).

## SUPPLEMENTAL MATERIAL

Supplemental material aem.00253-25-s0001.docx Tables S1 and S2; Fig. S1 to S4.

ASM does not own the copyrights to Supplemental Material that may be linked to, or accessed through, an article. The authors have granted ASM a non-exclusive, world-wide license to publish the Supplemental Material files. Please contact the corresponding author directly for reuse.
