# Supplementary material for: Vip3C proteins from Paenibacillus spp. for controlling lepidopteran crop pests
Source: Appl Environ Microbiol. 2025 Jun 20;91(7):e00253-25. doi: 10.1128/aem.00253-25 (PMC12285222; doi:10.1128/aem.00253-25)
Supplement: Supplemental material — Tables S1 and S2; Fig. S1 to S4. [file aem.00253-25-s0001.docx]

**Supplemental materials for Insecticidal proteins from *Paenibacillus* for control of Lepidoptera crop pests.**

**Supplemental Table 1**. Sequencing and assembly summary information about the genomes, including the output of BUSCO based on its autodectection of the lineage as Bacillales.

| **Genome** | **DSC004343** | **DSC020651** |
| --- | --- | --- |
| **Paired reads** | 10,529,014 | 10,193,684 |
| **Total assembly length** | 6,605,001 | 6,452,041 |
| **Scaffolds** | 171 | 235 |
| **Contigs** | 174 | 235 |
| **Gaps** | 0.003% | 0.000% |
| **N50** | 114 KB | 98 KB |
| **Complete BUSCOs** | 448 | 446 |
| **Complete & single-copy BUSCOs** | 442 | 439 |
| **Complete & duplicated BUSCOs** | 6 | 7 |
| **Fragmented BUSCOs** | 1 | 1 |
| **Missing BUSCOs** | 1 | 3 |
| **Total BUSCO groups searched** | 450 | 450 |

**Supplementary Table 2.** Cryo-EM data collection, refinement and validation statistics

|  | VIP3Cb1 Protoxin | VIP3Cb1 Toxin |
| --- | --- | --- |
|  | (PDB 9EFI) | (PDB 9EFG) |
|  | (EMD-47974) | (EMD-47972) |
| **Data collection and processing** |  |  |
| Microscope | Titan Krios G3 | Titan Krios G3 |
| Detector | Falcon 4 | Falcon 4 |
| Voltage (kV) | 300 | 300 |
| Magnification | 59,000 | 59,000 |
| Pixel size (Å) | 1.081 | 1.081 |
| Total electron dose (e/Å) | 57.6 | 53.3 |
| Exposure time (s) | 13.29 | 13.37 |
| Number of frames | 50 | 50 |
| Defocus range (µm) | -1 to -2.4 | -1 to -2.4 |
| Number of micrographs | 4,547 | 5,644 |
| Initial particles | 2,763,759 | 2,758,038 |
| Final particles | 1,557,854 | 219,620 |
| Symmetry imposed | C2 | C4 |
| Map Resolution (FSC 0.143) (Å) | 2.61 | 3.04 |
|  |  |  |
| **Refinement** |  |  |
| Composition (#) |  |  |
| Chains | 4 | 5 |
| Non-Hydrogen Atoms | 24710 | 21699 |
| Protein Residues | 3104 | 2708 |
| Ligand | 0 | 1 |
| Bonds (RMSD) |  |  |
| Length (Å) | 0.002 | 0.002 |
| Angles (°) | 0.423 | 0.444 |
| MolProbity score | 2.22 | 2.11 |
| Clash score | 8.47 | 6.92 |
| Ramachandran plot (%) |  |  |
| Outliers | 0.06 | 0.15 |
| Allowed | 7.06 | 6.13 |
| Favored | 92.88 | 93.72 |
| Rotomer outliers (%) | 2.58 | 2.61 |
| Mean ADP (B-factors) |  |  |
| Protein | 138 | 157 |
| Ligand | - | 30 |
| Map vs Model |  |  |
| CC (mask) | 0.80 | 0.82 |
| CC (box) | 0.76 | 0.85 |
| CC (peaks) | 0.69 | 0.77 |
| CC (volume) | 0.80 | 0.82 |
| Mean CC for ligands | - | 0.89 |


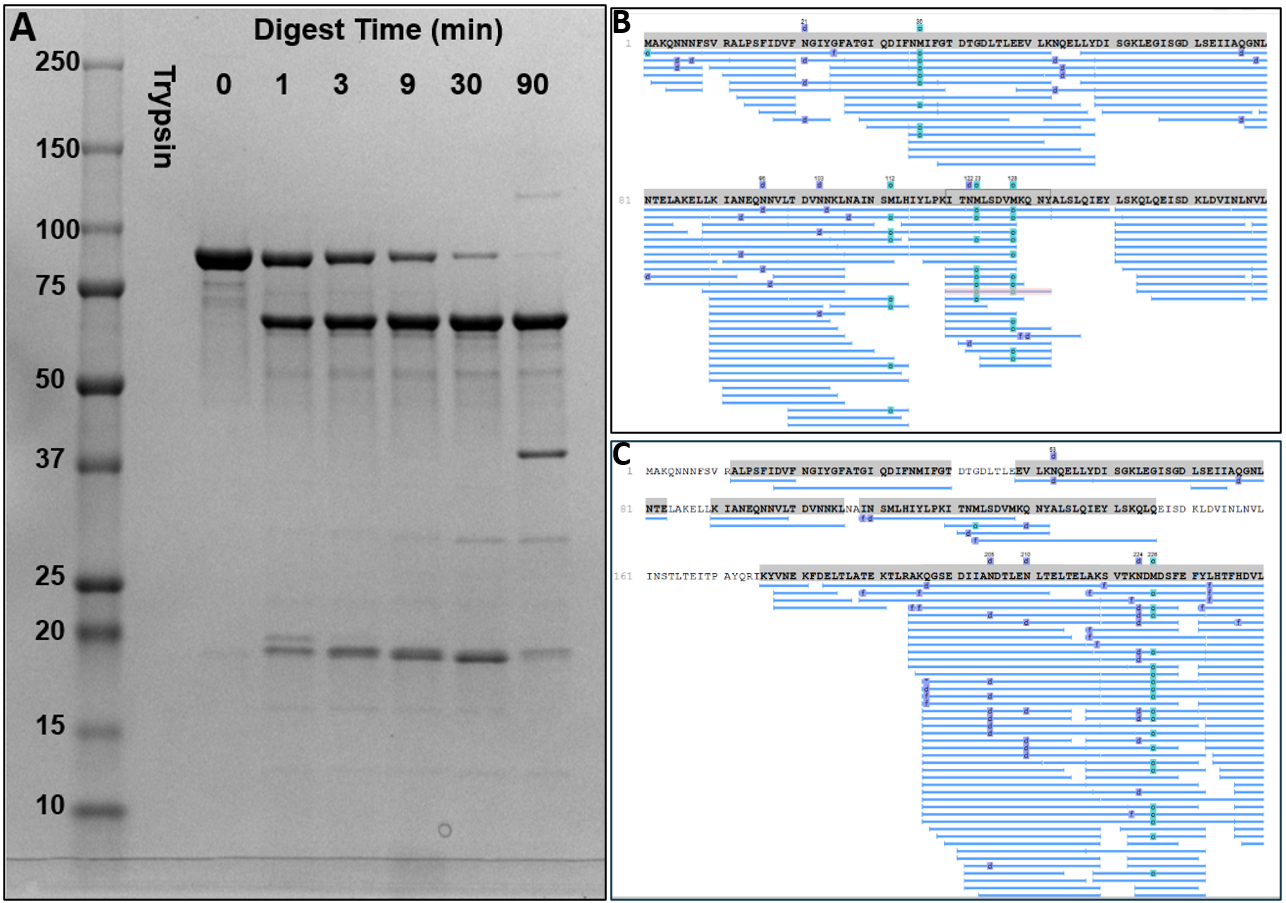


**Supplemental Figure 1. Trypsin time course and Mass Spectrometry to identify Vip3Cb1 N-terminus and activation site.** (A) Trypsin digest time course of Vip3Cb1 analyzed by SDS-PAGE (B) Vip3Cb1 protoxin peptide coverage when digested with chymotrypsin shows 100% peptide coverage. (C) Chymotryptic peptides observed from upper 68 kDa fragment of Vip3Cb1_act_ after 90 minutes of trypsin treatment showing a 2:1 preference for cleavage at Lysine195 over Arginine193.


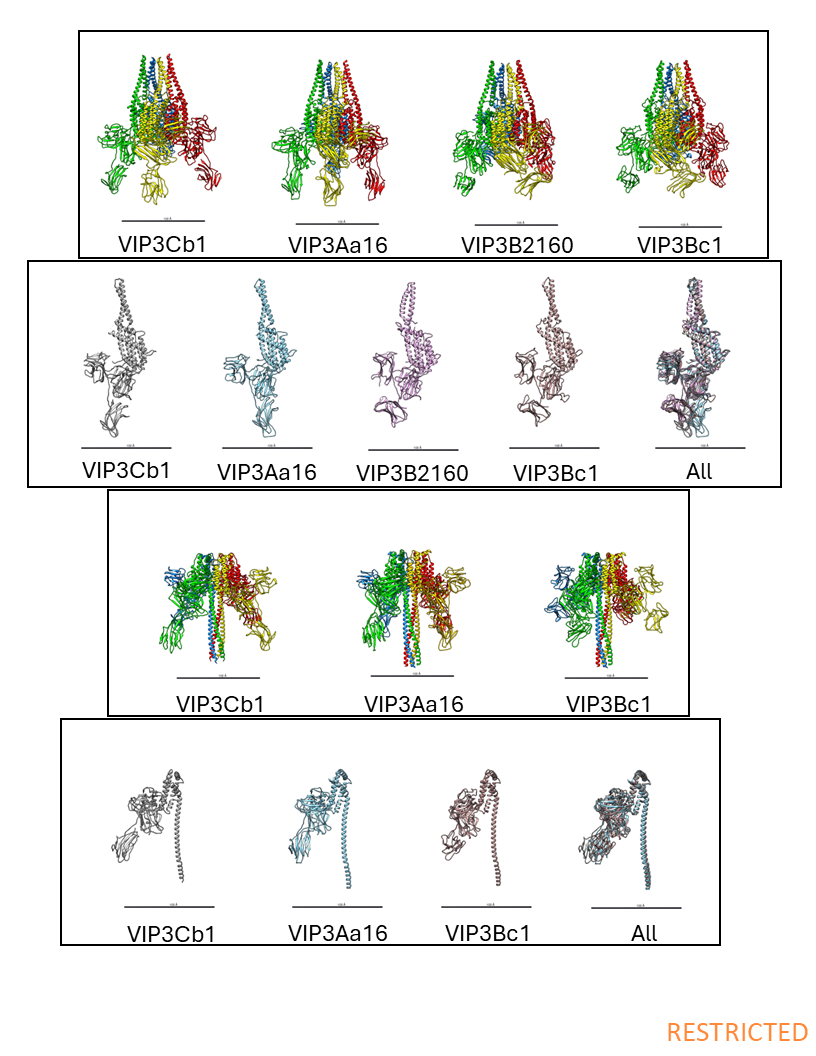


| **RMSD (Å)** | **VIP3Cb1.A** | **VIP3Aa16.A** | **VIP3Bc1.A** | **VIPB2160.A** |
| --- | --- | --- | --- | --- |
| **VIP3Cb1.A** | - | 0.987 | 1.094 | 1.094 |
| **VIP3Aa16.A** | 0.987 | - | 1.007 | 0.856 |
| **VIP3Bc1.A** | 1.094 | 1.007 | - | 1.019 |
| **VIPB2160.A** | 1.094 | 0.856 | 1.019 | - |

**Supplemental Figure 2. Structural comparison of Vip3Cb1 with other Vip3 structures.** Comparison of VIP3 structures determined to date by CryoEM (Vip3Cb1, Vip3Aa16, Vip3Bc1) and X-ray crystallography (Vip3B2160). Top two panels show protoxin tetramers (upper panel, colored as in Figure 4) and chain A monomers (lower panel) and superimposed (All). Lower two panels show activated toxin tetramers (upper panel, colored as in Figure 4) and chain A monomers (lower panel) and superimposed (All). Scale below each figure is 100 Angstroms. Table shows RMSD values (Å) calculated using UCSF Chimera’s Matchmaker for shown Vip3 structures’ Chain A.


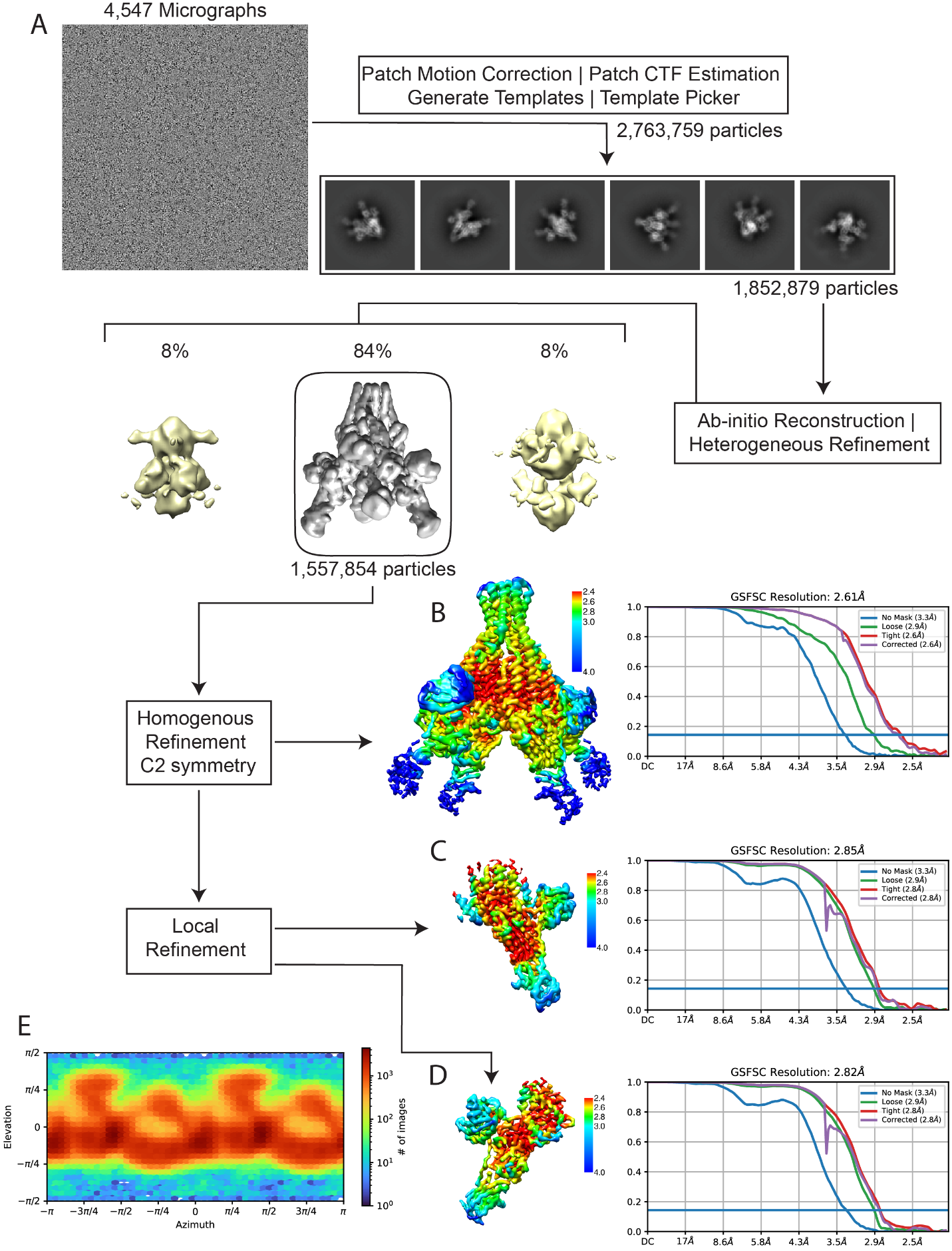


**Supplemental Figure 3**. **Cryo-EM data processing workflow for Vip3Cb1. A.** Representative micrograph of the Vip3Cb1 dataset containing 4,547 micrographs. Typical 2D class averages which were used to generate 3D density from heterogeneous refinement. Selected best class containing 1,557,854 particles as input for homogenous refinement imposing C2 symmetry. Followed by local refinement of the domains III-V for each chain of asymmetric unit was performed to improve alignment. **B.** Homogeneous refinement generated a 2.6Å map shown is the local resolution estimation map of homogenous refinement map and the gold-standard Fourier shell correlation (GSFSC) output from cryoSPARC. **C,D.** Local refinement generated 2.9Å and 2.8Å map respectively. Local resolution map shown along with the GSFCS output for each local refinement. **E.** Orientation distribution plot from cryoSPARC


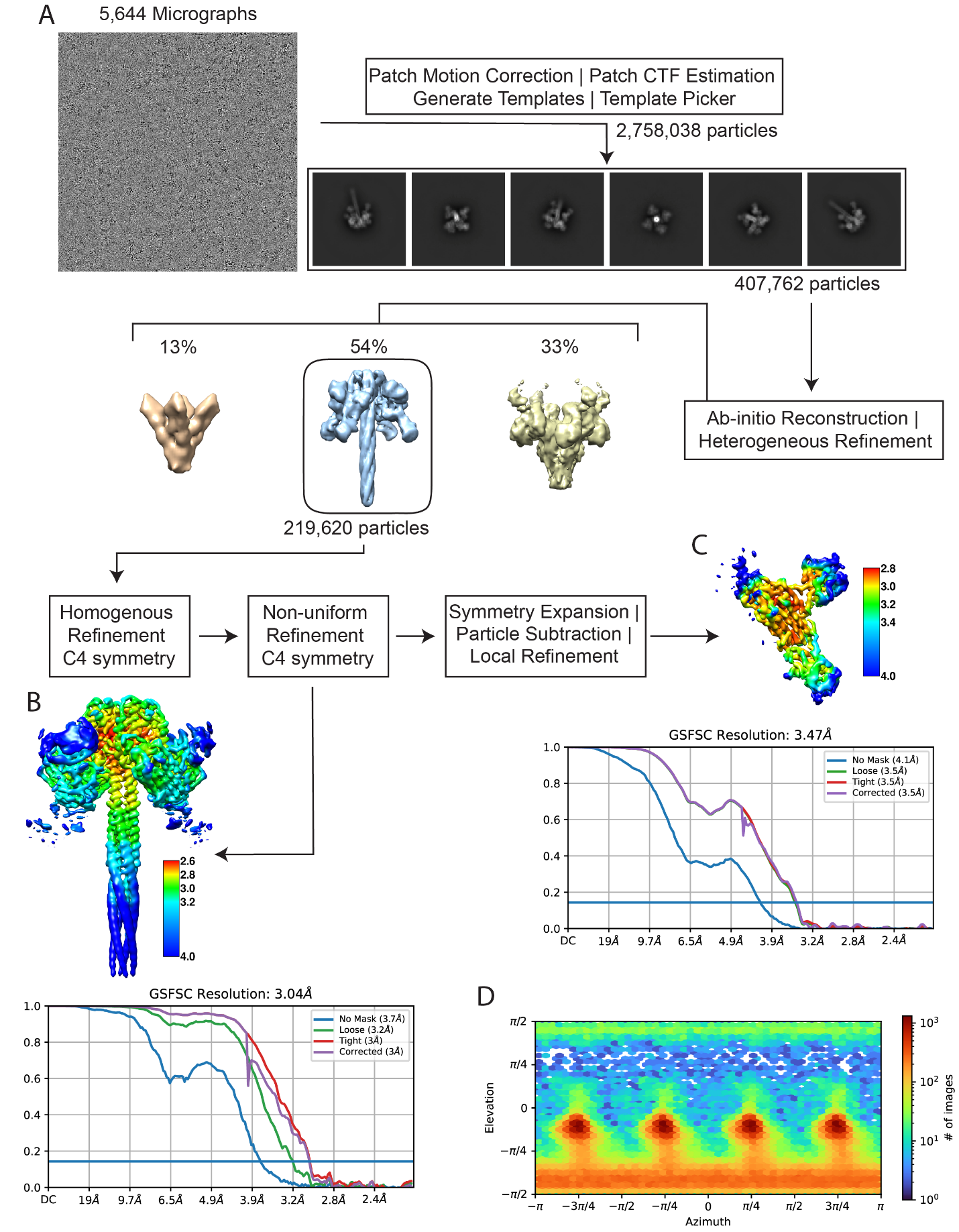


**Supplemental Figure 4**. **Cryo-EM data processing workflow for Vip3CB1 toxin. A.** Representative micrograph of the Vip3Cb1 dataset containing 5,644 micrographs. Typical 2D class averages which were used to generate 3D density from heterogeneous refinement. Selected best class containing 219,620 particles as input for homogenous refinement and subsequent non-uniform refinement both imposing C4 symmetry. Followed by local refinement after symmetry expansion and particle subtraction of the domains III-V improve alignment. **B.** Non-uniform refinement generated a 3.0 Å map shown is the local resolution estimation map of homogenous refinement map and the gold-standard Fourier shell correlation (GSFSC) output from cryoSPARC. **C.** Local refinement generated 3.5Å map and local resolution map shown along with the GSFCS output for each local refinement. **D.** Orientation distribution plot from cryoSPARC
